# Characterizing the transcriptome and microsatellite markers for almond (*Amygdalus communis* L.) using the Illumina sequencing platform

**DOI:** 10.1186/s41065-017-0049-x

**Published:** 2017-10-19

**Authors:** Linsen Zhang, Xiaoni Yang, Xiangning Qi, Chunhui Guo, Zhaobin Jing

**Affiliations:** College of Horticulture, Northwest A&F University, Yangling, 712100 Shaanxi People’s Republic of China

**Keywords:** *Prunus amygdalus* Batsch, Transcriptome, Functional classification, SSR markers

## Abstract

**Background:**

The almond tree (*Prunus amygdalus* Batsch) is an important nut tree grown in subtropical regions that produces nutrient-rich nuts. However, a paucity of genomic information and DNA markers has restricted the development of modern breeding technologies for almond trees.

**Results:**

In this study, almonds were sequenced with Illumina paired-end sequencing technology to obtain transcriptome data and develop simple sequence repeats (SSR) markers. We generated approximately 64 million clean reads from the various tissues of mixed almonds, and a total of 42,135 unigenes with an average length of 988 bp were obtained in the present study. A total of 27,586 unigenes (57.7% of all unigenes generated) were annotated using several databases. A total of 112,812 unigenes were annotated with the Gene Ontology (GO) database and assigned to 82 functional sub-groups, and 29,075 unigenes were assigned to the KOG database and classified into 25 function classifications. There were 9470 unigenes assigned to 129 Kyoto Encyclopaedia of Genes and Genomes (KEGG) pathways from five categories in the KEGG pathway database. We further identified 8641 SSR markers from 48,012 unigenes. A total of 100 SSR markers were randomly selected to validate quality, and 82 markers could amplify the specific products of *A. communis* L., whereas 70 markers were successfully transferable to five species (*A. ledebouriana, A. mongolica, A. pedunculata, A. tangutica,* and *A. triloba*).

**Conclusions:**

Our study was the first to produce public transcriptome data from almonds. The development of SSR markers will promote genetics research and breeding programmes for almonds.

**Electronic supplementary material:**

The online version of this article (10.1186/s41065-017-0049-x) contains supplementary material, which is available to authorized users.

## Background

The almond (*Prunus amygdalus* Batsch) is one of the world’s most important nut crops. The almond belongs to the genus *Prunus*, subgenus *Amygdalus* [1]. Almonds are widely used in food production and have high nutritional value [2]. Most *Amygdalus* wild species are highly tolerant of cold, drought and salt [3]. Almonds are a typical outbreeding species with gametophyte self-incompatibility [4]. They are also an antioxidant source [5] and an important germplasm resource in breeding programmes [6]. Because almonds have high nutritional value, past research has largely focused on physicochemical properties, seed nutrients and oil extraction [2, 7, 8]. However, despite high economic and nutritional values, genome and genetic resources regarding almonds are scarce, which has restricted the development of modern breeding technologies for almonds.

Molecular markers are important tools used in evaluating genetic diversity among plant species and plant molecular breeding (marker-assisted breeding). In almond trees, expressed sequence tags (ESTs) have been developed, but currently, there are only 3926 ESTs and gene loci available for almond in the NCBI GenBank database. Therefore, the number of ESTs is not sufficient to address almond tree molecular breeding development. Simple sequence repeats (SSRs) are widely used in studies on genetic diversity and relationships of plants because they are highly polymorphic, co-dominant and reproducible [9]. Moreover, SSRs markers could be used in the QTL mapping of important agronomical traits loci and marker-assisted selection in almond trees [10–13]. Presently, however, only a few SSRs have been reported in almonds [14–16]. SRAP and AFLP markers have been used to study genetic diversity and relationships in wild *Amygdalus* species [2]. A transcriptome is the complete collection of RNA that includes the full range of mRNA, tRNA, rRNA, and other noncoding RNA molecules expressed by one or a group of cells, organs or tissues in a particular environment or a specific developmental stage. The Illumina paired-end sequencing technique has been widely used for transcriptome analysis in plants [10, 17–19].

Transcriptome sequencing is helpful in developing SSR molecular markers, as it is reliable and efficient [20]. However, transcriptomic and associated molecular markers in almonds have not yet been reported. In this study, Illumina sequencing technology was used to analyse the transcriptome of almonds and develop EST-SSR markers. To our knowledge, this is the first study to characterize the almond transcriptome. Our study will provide a foundation for almond molecular biology and molecular breeding of almonds.

## Results

### Transcriptome generation and de novo assembly

**Fig. 1:**
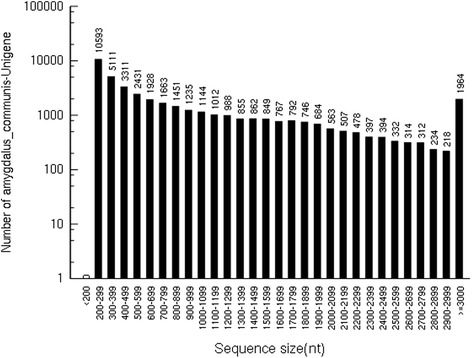
Length distributions of the unigenes. The x-axis indicates a different sequence size, and the y-axis indicates the unigene numbers of a specific sequence size

A total of 66,668,192 raw reads were generated from our Illumina HiSeq™ 2000 paired-end sequencing of almonds. The total length of the reads was approximately 10 Gigabase pairs (Gb); a total of 64,924,070 (97.38%) high-quality clean reads (1,440,780, 4.32%) as well as low-quality reads (1,702,602, 2.55%) were collected after removing the adapter. All high-quality reads were assembled using the Trinity program, and a total of 42,135 unigenes with an average length of 988 bp and an N50 length of 1714 bp were obtained. The unigenes ranged from 201 bp to 15,555 bp (Table 1). As shown in Fig. 1, 27,723 unigenes (65.80%) ranged from 201 to 1000 bp, 8699 unigenes (20.65%) were longer than 1000 bp and 5713 unigenes (13.56%) were longer than 2000 bp. The coverage percentage of read-blasted unigenes was 55.74% (more than 10 reads), 19.17% (more than 100 reads), and 25.09% (more than 1000 reads) (Additional file 1). All of the raw data were submitted to the NCBI database (accession number: PRJNA347906).

**Table 1:** Summary of transcriptome data for persimmon

Item	Number
1. Raw sequences and Assembly statistics	
Total reads	66,668,192
Total nucleotides (bp)	10,000,228,800
GC content percentage	46.77%
Total unigenes (average length; N50; min-max length)	42,135 (988; 1714; 201–15,555)
Mean RPKM value of unigenes (min-mix RPKM value)	14.8 (0–7109)
2. Bioinformatics annotations of mango fruit unigenes	
Gene annotation against Nr (%)	29,315 (69.6)
Gene annotation against Swiss-Prot (%)	20,642 (49.0)
Gene annotation against KEGG (%)	11,055 (26.2)
Gene annotation against KOG (%)	16,794 (39.9)
All annotation genes (%)	29,464 (69.9)
Without annotation genes (%)	12,671 (30.1)
GO Ontology (%)	
Biological process category	51,202;
Cellular component category	24,483;
Molecular function category	37,133;

### Functional annotation

**Fig. 2:**
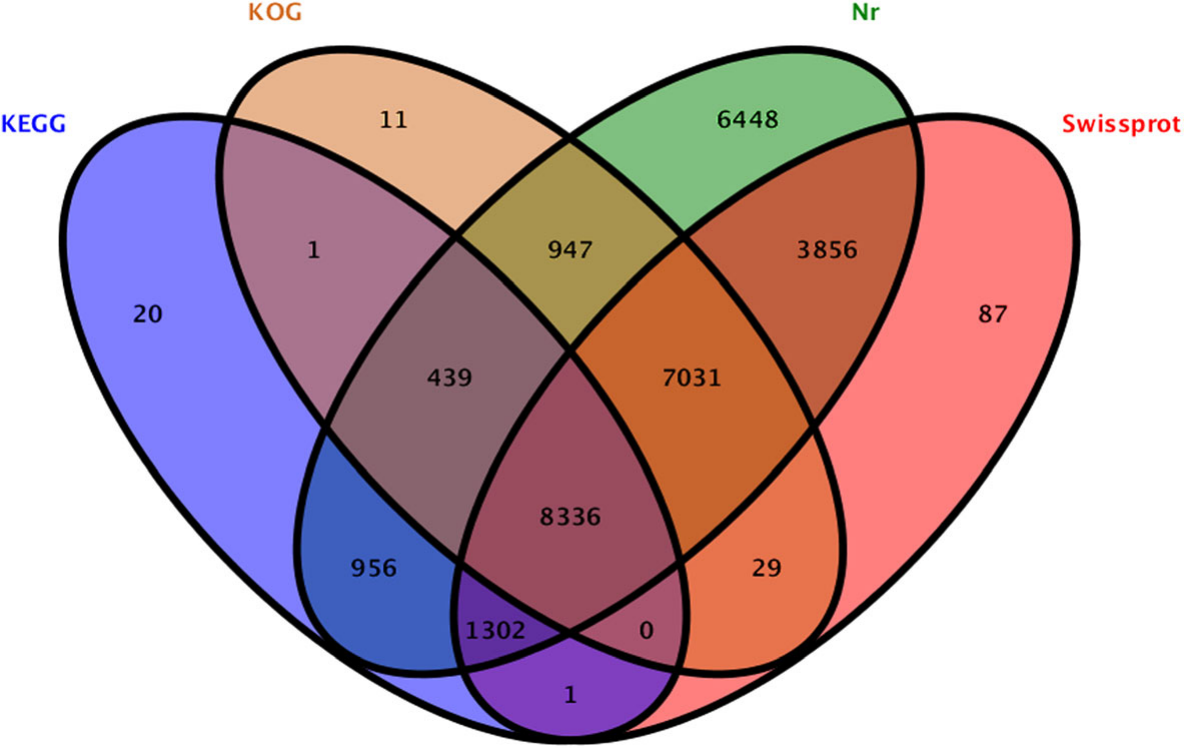
Venn diagram of Nr, Swiss-Prot, KOG and KEGG databases. Venn diagram showing homology sequence numbers of the unigenes in the Nr, Swiss-Prot, GOG and KEGG databases

The function annotation of the unigenes was performed in the Nr, Swiss-Prot, Kyoto Encyclopaedia of Genes and Genomes (KEGG) and KOG databases by BLASTX (E-value < 10^−5^). A total of 27,586 unigenes (57.7% of all unigenes) were annotated. A total of 29,315 unigenes (69.6% of all unigenes) of the largest match were annotated in the Nr database, followed by the Swiss-Prot (20,642, 49.0%), KOG (16,794, 39.9%) and KEGG (11,055, 26.2%) databases (Fig. 2). A total of 12,671 (42.5%) unigenes were not annotated in the four databases, indicating that these unigenes may be novel genes (Table 1). For the annotated unigenes in the Nr databases, the homologous sequences belonging to the species were analysed. The ten top-hit species were *Prunus mume* (18,097, 61.73%), *Malus domestica* (15,80, 5.40%), *Theobroma cacao* (1238, 4.22%), *Pyrus x bretschneideri* (1132, 3.86%), *Prunus persica* (1061, 3.46%), *Gossypium arboreum* (853, 2.90%), *Brassica napus* (710, 2.42%), *Medicago truncatula* (682, 2.33%), *Fragaria vesca subsp. vesca* (665, 2.27%), *Morus notabilis* (311, 1.06%), and others (3029, 10.34%).

### Functional classification by gene ontology (GO) and KOG

**Fig. 3:**
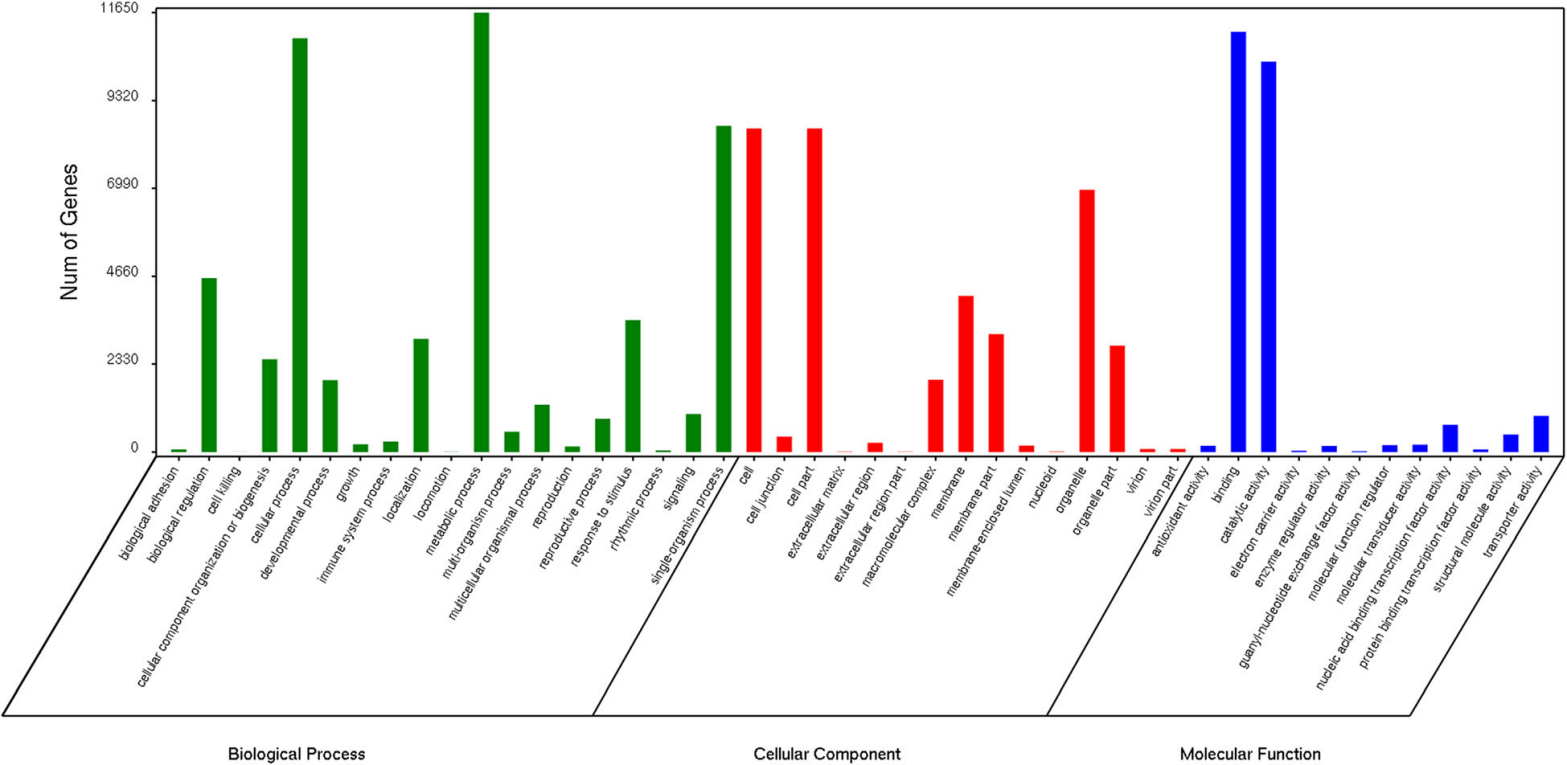
GO function classification of the almond transcriptome. There are three main GO categories: biological process, cellular component and molecular function. The x-axis indicates the categories, and the y-axis indicates the number of the unigenes

To further evaluate the functions of the almond unigenes, we used GO assignments to classify the almond unigene functions. A total of 112,812 unigenes were assigned to 82 functional sub-groups. Of the three ontology categories, the largest was biological process (51,202 unigenes), followed by cellular component (37,133 unigenes) and molecular function (24,483 unigenes) (Fig. 3). For the biological process group, the most frequent process was metabolic process (11,647, 22.75%), followed by cellular process (10,967, 21.42%). Cell (8581, 23.11%) and cell part (8581, 23.11%) were the most highly represented groups in the cellular component category. For the molecular function category, binding (11,139, 45.50%) and catalytic activity (10,346, 27.86%) represented the greatest proportion. The GO classifications of the unigenes are listed in Additional file 2.

**Fig. 4:**
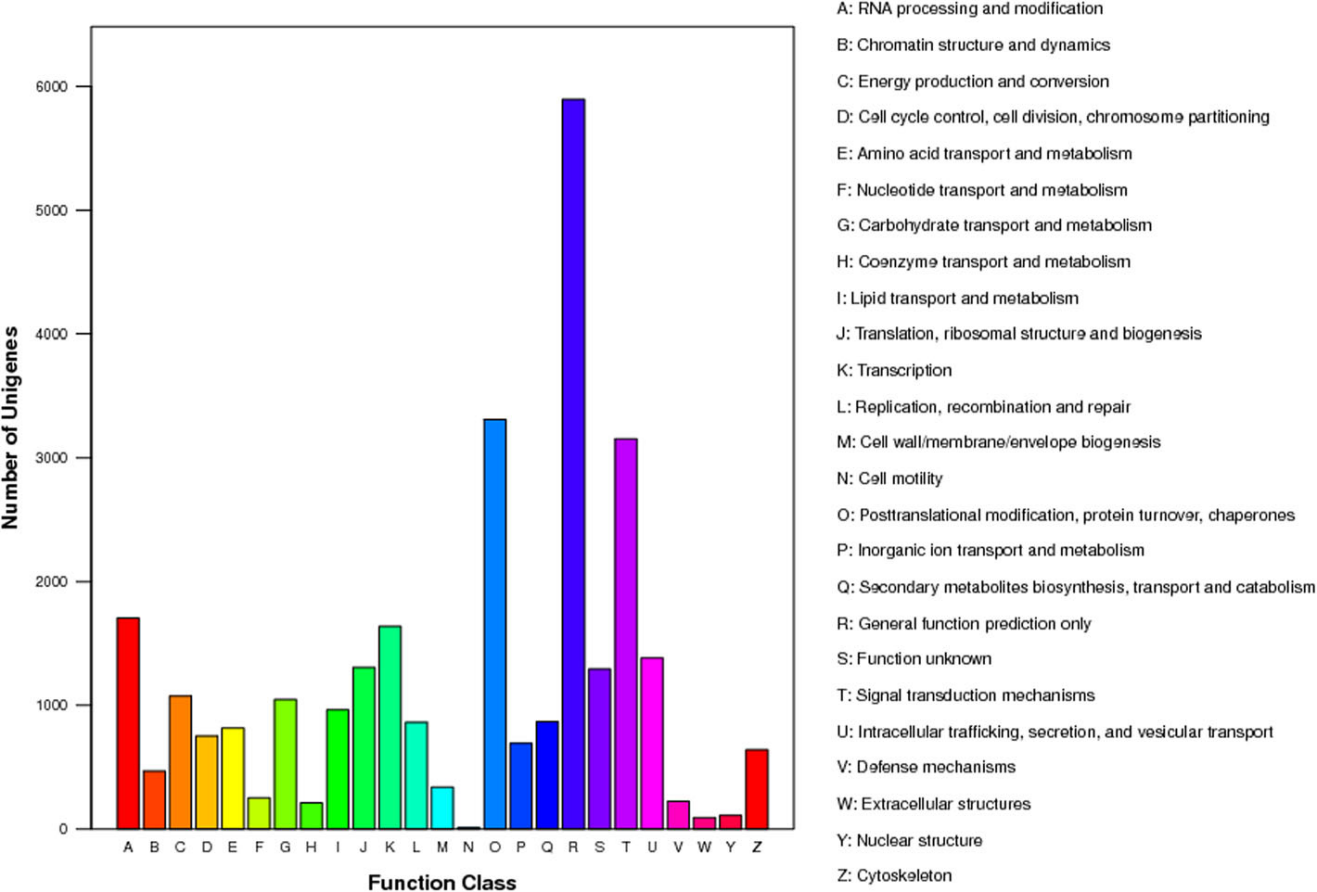
KOG function classification of the almond transcriptome. A total of 25 categories were obtained. The x-axis indicates the categories, and the y-axis indicates the numbers of the unigenes

KOG classifications were searched based on a BLAST search against the KOG database. A total of 29,075 unigenes were classified into 25 function classifications (Fig. 4). For the 25 KOG categories, the general function prediction was the largest group (5895, 20.28%); posttranslational modification, protein turnover, chaperones (3311, 11.39%), and signal transduction mechanisms (3152, 10.84%) had high percentages, and 15,012 unigenes were assigned to other functional categories (Additional file 3).

### Functional classifications by KEGG

The pathway annotations were used to analyse the biological functions of genes. In this study, 9470 unigenes were assigned to 129 KEGG pathways that belonged to five categories, namely, metabolic pathways (5648, 59.60%), genetic information processing (2636, 27.8%), cellular processes (512, 5.4%), environmental information processing (389, 4.1%) and organismal systems (285, 3.0%) (Additional file 4). The majority of the unigene pathways were associated with ribosomes (443, 7.3%), carbon metabolism (320, 5.3%), protein processing in the endoplasmic reticulum (290, 4.8%), biosynthesis of amino acids (277, 4.9%), spliceosomes (274, 4.5%), plant hormone signal transduction (249, 4.1%) and endocytosis (245, 4.0%).

### Development and characterisation of SSR markers

In this study, the unigene sequences were used to develop new SSR markers with MISA software. A total of 8641 SSRs were identified from 48,012 unigenes. For the 8641 SSRs, di-nucleotide motifs were the most abundant form (5141, 59.5%), followed by tri-nucleotides (2416, 28.5%), tetra-nucleotides (606, 7.0%), hexa-nucleotides (277, 3.2%) and penta-nucleotides (201, 2.3%) (Table 2). In addition, the number of repeated units of the di-nucleotide motifs ranged from 6 to 15, and the tri-nucleotide, tetra-nucleotide, penta-nucleotide, and hexa-nucleotide motifs included 5 to 15, 4 to 10, 4 to 8, and 4 to 7, respectively. SSRs with six tandem repeats were the most frequent (1795, 20.8%), followed by five tandem repeats (1543, 17.9%), more than fifteen tandem repeats (1228, 14.2%), seven tandem repeats (1095, 12.7%), and others (Table 3). The most frequent motif types of SSRs were AG/CT (49.0%), followed by AAG/CTT (9.3%), AT/AT (5.9%), AC/GT (4.5%), AGG/CCT (3.6%) and others (Additional file 5).

**Table 2:** Summary of the EST-SSRr data for almond

Item	Number
Total number of sequences examined	42,135
Total size of examined sequences (bp)	41,663,061
Total number of identified SSRs	8641
Number of SSR containing sequences	6780
Number of sequences containing more than 1 SSR	1433
Number of SSRs present in compound formation	752
Di-nucleotide	5141 (59.5%)
Tri-nucleotide	2416 (28.5%)
Tetra-nucleotide	606 (7.0%)
Penta-nucleotide	201 (2.3%)
Hexa-nucleotide	277 (3.2%)

**Table 3:** Summary of the different repeat units of identified EST-SSRs

Number of repeat unit	Di-	Tri-	Tetra-	Penta-	Hexa	Total	Percentage (%)
4	0	0	435	151	200	786	9.1
5	0	1321	121	39	62	1543	17.9
6	1156	592	31	7	9	1795	20.8
7	771	302	13	3	6	1095	12.7
8	596	67	4	1	0	668	7.7
9	566	38	1	0	0	605	7.0
10	439	58	1	0	0	498	5.8
11	237	11	0	0	0	248	2.9
12	38	9	0	0	0	47	0.5
13	5	10	0	0	0	15	0.2
14	109	4	0	0	0	113	1.3
≥ 15	1224	4	0	0	0	1228	14.2
Total	5141	2416	606	201	277	8641	100

### Cross-species transferability of *A. communis* SSR markers

**Fig. 5:**
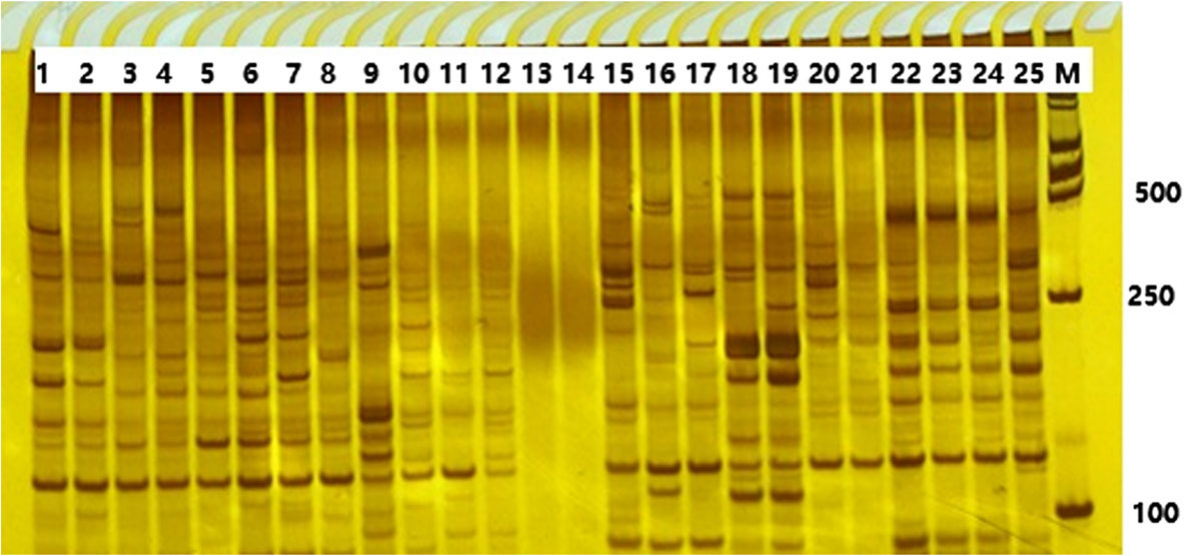
Examples of polymorphic products amplified by different SSR primer pairs. 1–25 represent different primer pairs. M represents DL2000 DNA markers

**Fig. 6:**
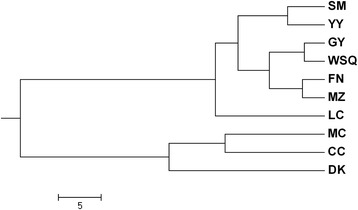
Dendrogram obtained using UPGMA cluster analysis based on Nei’s genetic distances among the ten populations of almonds. The population abbreviations are the same as those in Additional file 7

One hundred SSR sites were randomly selected to design SSR primers (Additional file 6). Among these 100 primer pairs, 82 could amplify the specific products (these 82 markers are highlighted in red in Additional file 6), while the remaining 18 did not generate PCR products. To validate the transferability of *A. communis* SSR markers, five species (*A. ledebouriana, A. mongolica, A. pedunculata, A. tangutica, and A. triloba*) (Additional file 7) were assessed using the 82 SSR markers selected above. The results indicated that 70 SSR markers were transferable to these five species and that 12 SSR markers did not generate bands. The PCR amplification results of some primers are shown in Fig. 5. The UPMGA cluster analysis indicated that *A. communis* and *A. mongolica* are more closely related (Fig. 6).

## Discussion

Almonds are one of the most important commercially cultivated crops in subtropical regions, specifically in southwest Asia, the Middle East, and the Mediterranean [8] because almonds have a high nutrient value. Previous studies concerning almonds focused on physicochemical properties, seed nutrients and oil extraction [2, 7, 8]. However, no studies have yet constructed a genetic linkage map, including QTL mapping of important agronomical traits and marker-assisted selection in almond trees. Until now, genome sequencing of some important fruits has been completed using NGS technologies. Transcriptome analysis based on NGS technologies, such as Illumina and 454 sequencing platforms, has provided an efficient tool for obtaining genomic data for some plants without a reference genome, such as wax gourds [21] and pumpkins [22]. Therefore, we sequenced the almond transcriptome to obtain genomic data and then developed many SSR markers. These transcriptome data will provide information for future studies on breeding and molecular biology.

In this study, a large number of transcriptomic unigenes (42135) was obtained using the Illumina HiSeq^TM^ 2500 platform, and the average unigene length was 988 bp. Consistent with recently published plant species, the average length of the unigenes was relatively long (835 bp) compared to black gram (443 bp) [10], caragana (709 bp) [18], wax gourd (709 bp) [21], pumpkin (765 bp) [22], Siberian apricot (652 bp) [23], Chinese jujube (473.4 bp) [24], and safflower (446 bp) [25]. These results indicate a higher quality of almond transcriptome sequencing and de novo assembly. In this study, approximately 27,586 unigenes (57.7% of all unigenes) were annotated by BLAST searches of the Nr, GO, Swiss-Prot, KEGG and KOG databases. Moreover, 12,671 (42.5%) of the unigenes did not annotate to any databases. Technical limitations, such as read length and sequencing depth, may account for these unannotated unigenes. For gene annotation, the sequences of unigenes were blasted against the Nr, Swiss-Prot, KEGG, GO and KOG databases. Approximately 27,586 unigenes (57.7% of all unigenes) were annotated in four protein databases, indicating that the transcriptomic data of almonds may have large transcript diversity. Additionally, approximately 12,671 (42.5%) unigenes were not annotated to the four databases, suggesting that some unigenes may be unique to almonds.

SSR markers have been important in some research, including the assessment of genetic diversity and genetic relationships, the construction of genetic maps, marker-assisted selection of important agronomic traits, and others [2, 26]. Previous studies have also shown that SSR markers are highly polymorphic, codominant and easily reproducible [9]. Due to the time-consuming and expensive nature of traditional methods for SSR marker development, few SSR markers have been reported for almonds, and no studies have reported the development of SSR markers in *A. communis*, which has limited the application of SSR markers in almond trees. Transcriptome sequencing is an efficient technology for the development of SSR markers in plants, and the SSR markers for some plants have been reported using transcriptome sequencing [10, 21–25]. Our results produced a large number of transcriptome sequences that could be used to develop SSR markers in almonds. In total, 8641 SSR markers were identified from 48,012 unigenes. In this study, di-nucleotide motifs were the most abundant form, followed by tri-nucleotides, tetra-nucleotides, hexa-nucleotides and penta-nucleotides, which is similar to previous studies [23–25]. In addition, the most abundant di-nucleotides and tri-nucleotides were AG/CT and AAG/CTT, respectively, which was consistent with previous reports [20–24]. To assess the quality of SSR markers, we randomly selected 100 pairs of primers and assessed them in five species. Eighty-two percent showed polymorphisms. This result was similar to results found in other plants. The UPMGA cluster analysis indicated that *A. communis* and *A. mongolica* were more closely related, which was consistent with Jing et al., who reported using SRAP markers [2]. We believe that the new SSR markers will be used to study genetic diversity, genetic mapping, and, in particular, marker-assisted breeding for almonds.

## Conclusion

This paper reports on the transcriptome characterizations of almond trees and provides a large number of SSR markers to elucidate the molecular biology of almond trees. To our knowledge, this is the first attempt to develop SSR markers for almonds using a transcriptome sequencing method, and these developed SSR markers will significantly contribute to genetic diversity studies, QTL mapping, and marker-assisted selection breeding for almonds. Notably, due to high transferability, these SSR markers may provide an efficient tool to accelerate molecular breeding in other *Amygdalus* species.

## Methods

### Plant materials and RNA extraction

Plants of *A. communis* were grown at the experimental farm of Northwest A&F University, Yangling, China. Tissues from leaves, flowers, stems and fruits were harvested from six individuals. The sampled tissues were immediately frozen in liquid nitrogen and stored at −80 °C for later RNA extraction. The RNA samples were isolated using an E.Z.N.A.® Plant RNA Kit (Omega Bio-tek, Inc.) according to the manufacturer’s protocol. The quality and quantity of RNA were assessed using electrophoresis on 1% agarose gels and a NanoDrop 1000 spectrophotometer (Thermo Scientific, Wilmington, DE, USA), respectively. High-quality RNA was used for further analyses. Equal amounts of RNA from different samples were pooled for further studies.

### cDNA library construction and transcriptome sequencing

After the RNA was extracted, the cDNA library construction was performed using a TransCript® cDNA sample prep kit (TransGen Biotech, China). The ligation products were size-selected with agarose gel electrophoresis, PCR-amplified, and sequenced using Illumina HiSeqTM 2500 by Gene Denovo Biotechnology Co., Ltd. (Guangzhou, China).

### Data filtering, de novo assembly and function annotation

Reads obtained from the sequencing machines included raw reads containing adapters or low-quality bases that would affect the following assembly and analysis. Thus, to obtain high-quality clean reads, the clean reads were assembled using the Trinity assembly program [27]. The isoform was obtained using Trinity software. To eliminate redundant information, the longest isoform was taken as the gene to further analyse; this was defined as the unigene. The functional annotation of unigene sequences was performed by BLASTX search of the non-redundant (Nr) (http://www.ncbi.nlm.nih.gov) database, Swiss-Prot protein database, Kyoto Encyclopaedia of Genes and Genomes (KEGG) database (http://www.genome.jp/kegg), Clusters of Orthologous Groups (KOG) database, and Gene Ontology (GO) database with an E-value < 10–5. We used the Blast2GO program to analyse the GO annotations of the unigenes [28]. The functional classifications were determined with WEGO software [29]. Pathway information of unigenes was collected from KEGG databases [30].

### SSR detection and primer design

MISA software (http://pgrc.ipk-gatersleben.de/misa/misa.html) was used to identify microsatellites in the whole transcriptome. The parameters were as follows: definition (unit_size, min_repeats): 2–6 3–5 4–4 5–4 6–4; interruptions (max_difference_between_2_SSRs): 100. If the distance between two SSRs was shorter than 100 bp, they were considered to be one SSR. Based on the MISA results, primer pairs of each SSR loci were designed using Primer premier 3.0 (PREMIER Biosoft International, Palo Alto, CA) in the flanking regions of SSRs.

### Validation of SSR markers

To validate the SSR markers, a total of 100 primer pairs were randomly selected and synthesized. Wild almond germplasm from six species (*A. communis, A. ledebouriana, A. mongolica, A. pedunculata, A. tangutica, and A. triloba*) were used to validate the SSR markers. The total genomic DNA was extracted from fresh leaves using a plant DNA extraction kit (TIANGEN^®^, China). The DNA quality and concentration were tested with a NanoDrop ND 1000 spectrophotometer (Thermo Scientific, USA). PCR amplification reactions were performed in a 25 μL volume, containing 40 ng of DNA, 0.2 mM dNTPs, 1.5 pM aliquots of forward and reverse primers, 2.5 mM Mg^2+^, 1 U Taq DNA polymerase (TaKaRa Biotechnology Dalian Co., Ltd., China), and 1× Taq Buffer (10 mM Tris-HCl, pH 8.3, 50 mM KCl). PCR amplification was performed with the following conditions: initial denaturation at 94 °C for 5 min; 30 cycles at 94 °C for 30 s, a primer-specific annealing temperature for 60 s, and 72 °C for 90 s; and 72 °C for 7 min. PCR products were separated by electrophoresis on denaturing 6% polyacrylamide gels and visualized using silver staining. The molecular size of the amplified fragments was estimated using a 10-bp DNA ladder (TransGen Biotech, China).

## Additional files

Additional file 1: The coverage percentage of reads blasted unigenes. (PNG 25 kb)

Additional file 2: Frequency distribution of SSRs from different motif types. (XLSX 11 kb)

Additional file 3: The gene ontology classification of assembled unigenes. (XLSX 10 kb)

Additional file 4: The details of KOG classifications. (XLSX 14 kb)

Additional file 5: The 129 KEGG pathway annotations. (PNG 11 kb)

Additional file 6: SSR sites developed in this study. (XLSX 1402 kb)

Additional file 7: List of species in this study. (DOCX 16 kb)

## Abbreviations

GO: Gene Ontology
KEGG: Kyoto Encyclopaedia of Genes and Genomes
KOG: Clusters of Orthologous Groups
Nr: non-redundant
SSR: simple sequence repeats marker
