# Supplementary figures and images for: Characterizing the transcriptome and microsatellite markers for almond (Amygdalus communis L.) using the Illumina sequencing platform
Source: Hereditas. 2017 Oct 19;155:14. doi: 10.1186/s41065-017-0049-x (PMC5649074; doi:10.1186/s41065-017-0049-x)

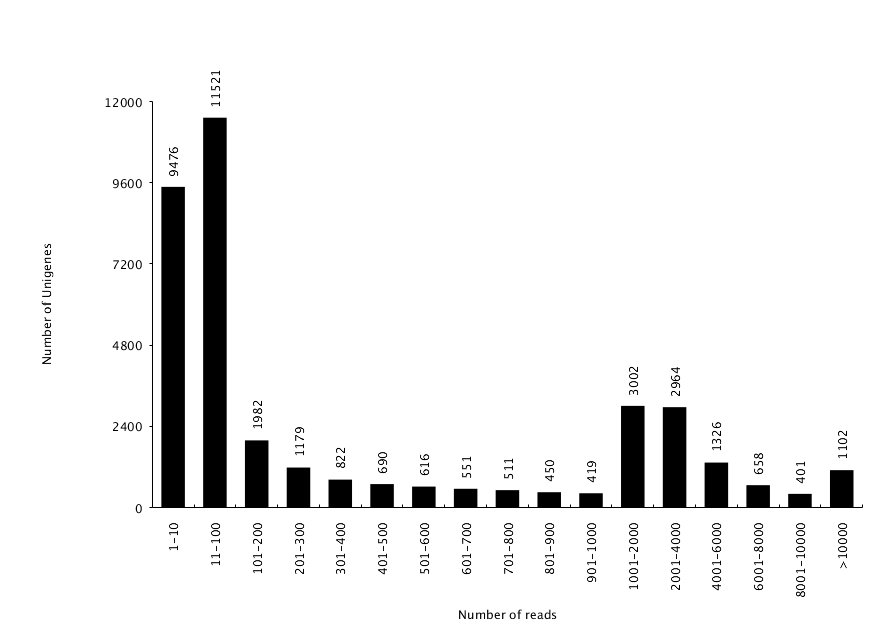

Supplement: Supplementary file 1 — The coverage percentage of reads blasted unigenes. (PNG 25 kb) [file 41065_2017_49_MOESM1_ESM.png]

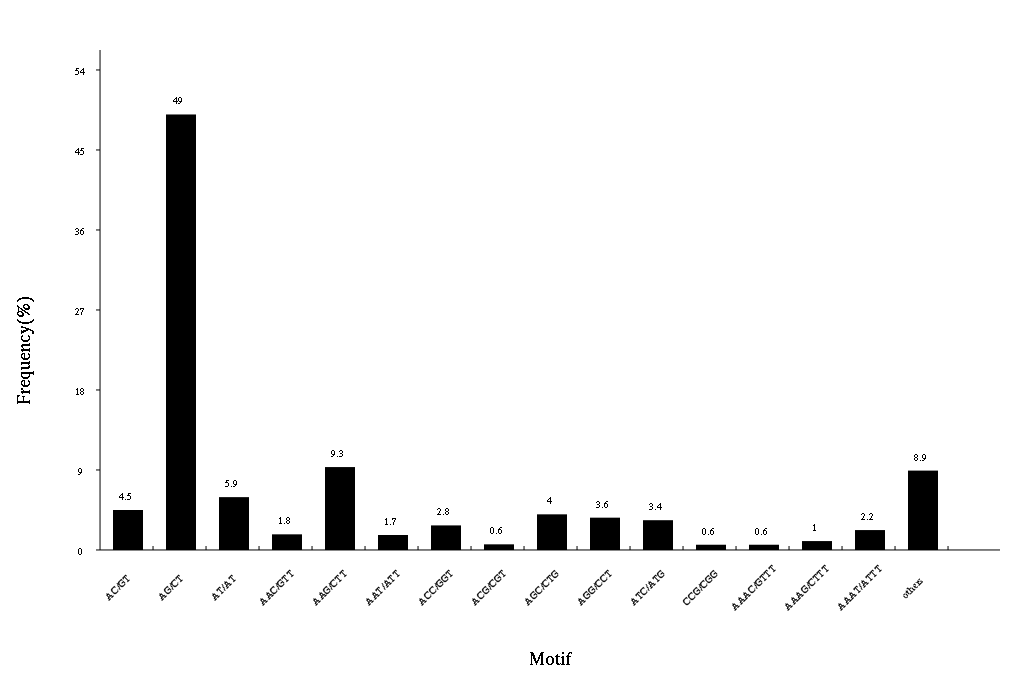

Supplement: Supplementary file 5 — The 129 KEGG pathway annotations. (PNG 11 kb) [file 41065_2017_49_MOESM5_ESM.png]
