# Supplementary material for: Characterizing the transcriptome and microsatellite markers for almond (Amygdalus communis L.) using the Illumina sequencing platform
Source: Hereditas. 2017 Oct 19;155:14. doi: 10.1186/s41065-017-0049-x (PMC5649074; doi:10.1186/s41065-017-0049-x)
Supplement: Supplementary file 7 — List of species in this study. (DOCX 16 kb) [file 41065_2017_49_MOESM7_ESM.docx]

**Table 1** Sampling locations and geographic distribution of almond in this study.

| Species | | Location | Population  code | Sample code | Altitude (m) | Longitude (E) | Latitude (N) | AMT (℃) | AMP (mm) |
| --- | --- | --- | --- | --- | --- | --- | --- | --- | --- |
| *A. pedunculata* Pall. | | Shenmu County, Shaanxi Province | SM | 1-11 | 1199 | 110.167783 | 38.745492 | 8.5 | 440.8 |
|  |  | Yuyang County, Shaanxi Province | YY | 12-22 | 1215 | 109.118272 | 38.358217 | 8.1 | 407.0 |
|  |  | Fengning County, Hebei Province | FN | 23-31 | 1390 | 116.350167 | 41.961774 | 7.9 | 506.3 |
|  |  | Guyang County, Inner Mongolia Autonomous Region | GY | 32-41 | 1550 | 110.386269 | 40.92095 | 4.0 | 300.0 |
|  |  | Wushenqi County, Inner Mongolia Autonomous Region | WSQ | 42-51 | 1301 | 108.845906 | 38.592428 | 7.0 | 370.5 |
| *A. triloba* (Lindl) Ricker. | | Mizhi County, Shaanxi Province | MZ | 52-65 | 860 | 110.171247 | 37.749833 | 8.5 | 451.6 |
| *A. tangutica Batal*. | | Li County, Gansu Province | LC | 66-73 | 1420 | 105.180003 | 34.187625 | 9.9 | 500 |
| *A. mogolica Maxim.* | | Dengkou County, Inner Mongolia Autonomous Region | DK | 74-81 | 1041 | 106.747406 | 40.700217 | 7.6 | 143.9 |
| *A. communis* L. | Meixin | Mei County, Shaanxi Province | MC | 82-87 | 514 | 107.750258 | 34.273561 | 12.9 | 589 |
|  | Chaomei | Chengcheng County, Shaanxi Province | CC | 88-95 | 558 | 109.939278 | 34.979125 | 12.0 | 546.4 |

AMT: annual mean temperature; AMP: annual mean precipitation.
